# Ablating Adult Neurogenesis in the Rat Has No Effect on Spatial Processing: Evidence from a Novel Pharmacogenetic Model

**DOI:** 10.1371/journal.pgen.1003718

**Published:** 2013-09-05

**Authors:** James O. Groves, Isla Leslie, Guo-Jen Huang, Stephen B. McHugh, Amy Taylor, Richard Mott, Marcus Munafò, David M. Bannerman, Jonathan Flint

**Affiliations:** 1 The Wellcome Trust Centre for Human Genetics, The University of Oxford, Oxford, United Kingdom; 2 Department and Graduate Institute of Biomedical Sciences, College of Medicine, Chang Gung University, Tao-Yuan, Taiwan; 3 The Department of Experimental Psychology, The University of Oxford, Oxford, United Kingdom; 4 The School of Experimental Psychology, University of Bristol, Bristol, United Kingdom; University of Chicago, United States of America

## Abstract

The function of adult neurogenesis in the rodent brain remains unclear. Ablation of adult born neurons has yielded conflicting results about emotional and cognitive impairments. One hypothesis is that adult neurogenesis in the hippocampus enables spatial pattern separation, allowing animals to distinguish between similar stimuli. We investigated whether spatial pattern separation and other putative hippocampal functions of adult neurogenesis were altered in a novel genetic model of neurogenesis ablation in the rat. In rats engineered to express thymidine kinase (TK) from a promoter of the rat glial fibrillary acidic protein (GFAP), ganciclovir treatment reduced new neurons by 98%. GFAP-TK rats showed no significant difference from controls in spatial pattern separation on the radial maze, spatial learning in the water maze, contextual or cued fear conditioning. Meta-analysis of all published studies found no significant effects for ablation of adult neurogenesis on spatial memory, cue conditioning or ethological measures of anxiety. An effect on contextual freezing was significant at a threshold of 5% (P = 0.04), but not at a threshold corrected for multiple testing. The meta-analysis revealed remarkably high levels of heterogeneity among studies of hippocampal function. The source of this heterogeneity remains unclear and poses a challenge for studies of the function of adult neurogenesis.

## Author Summary

Adult neurogenesis occurs in the rodent brain, but its function remains unclear. Current theories support the view that adult neurogenesis in the hippocampus supports pattern separation in the hippocampus, thereby allowing animals to distinguish between similar, overlapping inputs. However the effects of pharmacological, radiation and genetic ablation of adult neurogenesis on putative hippocampal functions have been inconsistent. We developed a novel genetic model to ablate adult neurogenesis in the rat. We found that we could reduce adult neurogenesis by 98%. Rats without adult neurogenesis showed no significant difference from controls in learning and memory tasks nor spatial pattern separation. We investigated the sources of heterogeneity in published results using a meta-analysis. The source of this heterogeneity remains unclear and poses a challenge for studies of the function of adult neurogenesis.

## Introduction

Adult neurogenesis occurs in the olfactory bulb and the hippocampus, where stem-cell like progenitor cells proliferate throughout adult life to generate functionally active neurons [1], [2], [3], [4], [5], [6], [7]. Whether this cellular population has a specific role in hippocampal processing, or is even necessary for normal emotional and cognitive functioning, remains controversial.

While some groups have shown that decreasing adult neurogenesis in the mouse can increase anxiety [8], [9] others report that the same behavioral effect is seen in rats following an increase in cell proliferation [10]. Numerous studies report that neither increasing nor decreasing adult neurogenesis has any impact on emotional behavior [11], [12], [13], [14], [15], [16]. The literature regarding the role of adult neurogenesis in spatial and contextual learning tells an equally confusing story. Reducing cell proliferation in rats has been reported to cause a deficit in both water maze performance [17], [18], [19], [20], [21] and contextual fear conditioning [22], [23], [24], [25]. But, again, others studies report no such effects [13], [18], [20], [22], [26].

The conflicting findings and hypotheses as to what adult neurogenesis does may, in part, reflect heterogeneity of experimental design. Adult neurogenesis has been ablated in a number of ways (genetic, irradiation and chemical means) that differ in efficacy, side effects and the specific cells affected [21], [27], [28]. Results obtained from mice and rats might vary because of differences in the properties of adult-born neurons between these two species [29], [30], [31], or because of other relevant species differences, such as the levels of stress and arousal experienced. Results may also vary because of differences in test protocols. For example, it has been suggested that adult neurogenesis might affect only remote memory, which is not measured in all studies [32]. It may also be the case that the behavioral paradigms currently used, either completely or in part, fail to test the neuronal mechanisms and psychological processes affected by adult neurogenesis.

Recently it has been argued that the effect of ablating adult neurogenesis on spatial memory can be attributed to pattern separation ability. Pattern separation is the ability to distinguish between similar, overlapping inputs [33], [34]. For example, in a spatial pattern separation task on the radial maze an animal might be presented with the choice of two goal arms, only one of which leads to a food reward. It has been suggested that when pattern separation is compromised it is more difficult for an animal to distinguish between goal arms that are close together, and therefore share a number of environmental, extra-maze spatial cues, than between two arms that are far apart and therefore more distinct. Reports now suggest that inhibiting adult neurogenesis impairs pattern separation [35], [36], [37], while increasing proliferation improves pattern separation [38], [39]. Thus, it is claimed that pattern separation is specifically the task of young adult-born granule cells in the hippocampus [40].

The finding that adult neurogenesis has been identified in all mammals in which it has been assessed, combined with the presumed conservation of function of the hippocampus in mammals, led us to hypothesize that the role of neurogenesis in pattern separation would be robust to the species in which it is assessed and to the test used for its assessment. The effect of ablating neurogenesis on pattern separation has so far only been tested in mice, so we developed and behaviorally characterised a novel genetically engineered rat model for the inducible inhibition of adult neurogenesis. We assessed emotional and cognitive phenotypes, including pattern separation. We show that adult neurogenesis is not essential for hippocampus-dependent spatial pattern separation. In addition, we carried out a comprehensive quantitative analysis of the literature (a meta-analysis) to identify sources of heterogeneity that might explain these results.

## Results

### Generation of a GFAP-TK rat

In the GFAP-TK pharmacogenetic approach for inhibiting adult neurogenesis, a virus thymidine kinase (TK) is expressed from the promoter of the glial fibrillary acidic protein (GFAP) gene, and therefore in all GFAP-positive cells. The antiviral drug ganciclovir (GCV) is metabolised by the TK enzyme into toxic nucleoside analogues resulting in the cell-specific termination of DNA synthesis. As GFAP is expressed in type-1 progenitor cells, which give rise to adult-born neurons in the subgranular zone of the dentate gyrus, and the subventricular zone of the olfactory bulb, this approach allows the selective, inducible inhibition of adult neurogenesis [13], [41].

**Figure 1:**
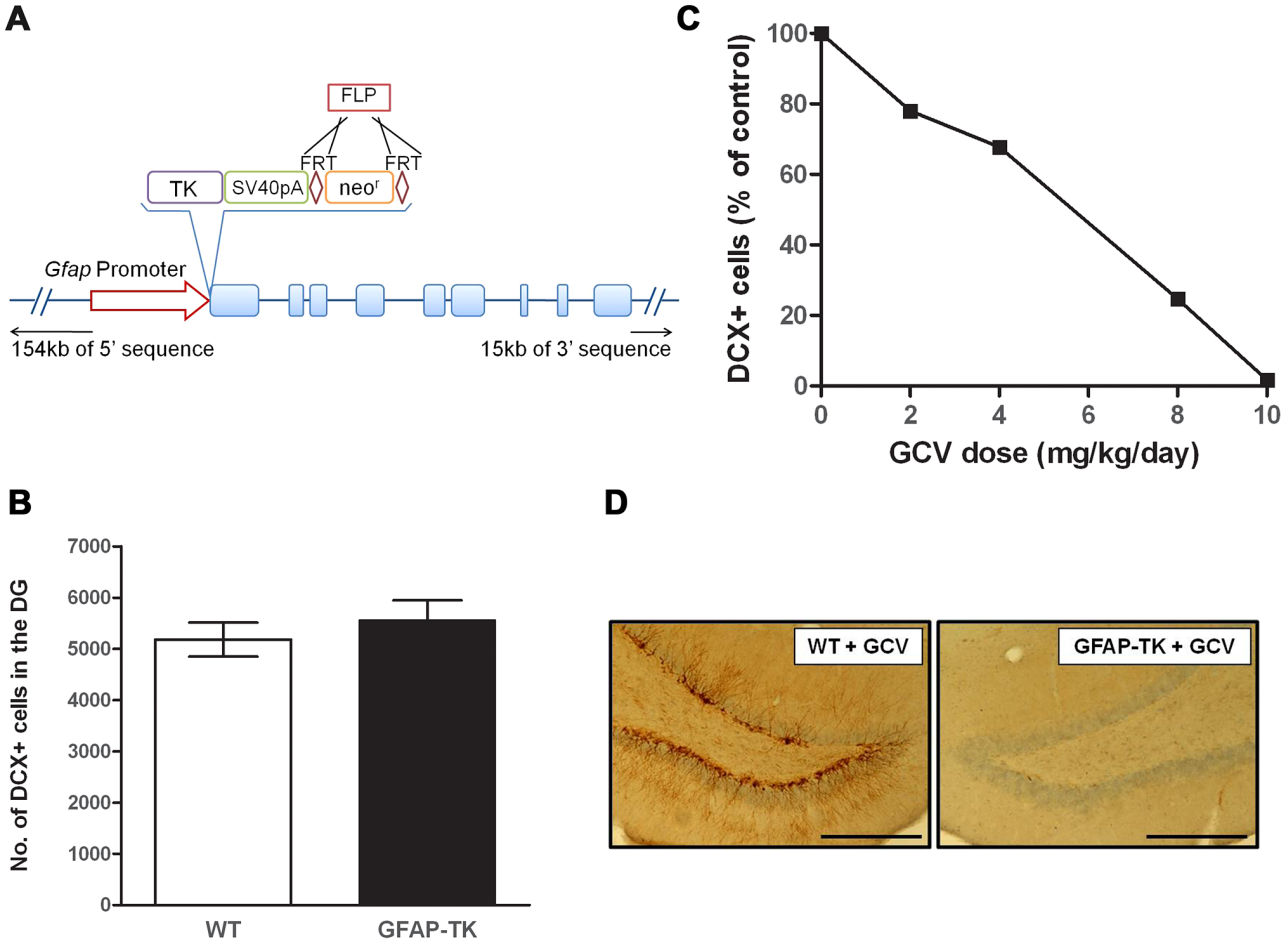
Ganciclovir administration ablates neurogenesis in the dentate gyrus of the GFAP-TK rat. **A**, Schematic of the *Tk*-pA-FRTneo^r^FRT genetic construct and position of insertion to replace the start codon of the rat *Gfap* gene within a Bacterial Artificial Chromosome (BAC). **B**, Number of doublecortin (DCX) positive (+) cells in the dentate gyrus of untreated 7-week old wild type (n = 5) and GFAP-TK (n = 5) rats. Data expressed as mean DCX+ cell counts (± s.e.m), generated from four 40 µm coronal sections taken from a ‘1 in 8’ series, starting at −2.5 mm from Bregma, along the dorsal/ventral extent of the hippocampus [73]. **C**, Number of DCX+ cells in the dentate gyrus of GFAP-TK rats dosed for 28 days with vehicle (saline, n = 2), or Ganciclovir (GCV) at 2 mg/kg/day (n = 2), 4 mg/kg/day (n = 2), 8 mg/kg/day (n = 2), or 10 mg/kg/day (n = 2). Data expressed as mean DCX+ cells as a percentage of vehicle control, generated from eight 40 µm coronal sections taken from a ‘1 in 12’ series from −2.5 mm from Bregma. **D**, Example of a DCX-stained dentate gyrus section from a wild type and GFAP-TK rat chronically treated with GCV (28 days, 10 mg/kg/day). Scale bar represents 200 µm.

We generated a GFAP-TK rat, analogous in construction to the GFAP-TK mouse. However while the mouse transgene contains only 2.2 kb of 5′ sequence and 1.4 kb of 3′ sequence (sufficient to drive endogenous GFAP-like expression of LacZ in mice [42]), the rat GFAP-TK model was generated by engineering a rat bacterial artificial chromosome that, in addition to the entire *Gfap* gene, contains an additional 154 kb of 5′, and 15 kb of 3′ sequence. Incorporation of additional flanking sequence is expected to confer tissue specific expression on the construct [43]. Figure 1a illustrates the construct design.

### Chronic GCV abolishes adult neurogenesis in the GFAP-TK rat

There was no difference in the basal levels of neurogenesis between untreated GFAP-TK and wild type controls (wild type n = 5, GFAP-TK n = 5; two-sample unpaired t-test; t(8) = 0.8, P = 0.47), indicating that the expression of the transgene alone does not affect cell proliferation (Figure 1b). Systemic administration of GCV to GFAP-TK rats for 4 weeks decreased the number of DCX-positive cells in the dentate gyrus in a dose-dependent manner, with the highest dose of 10 mg/kg/day causing a 98.3% reduction compared with vehicle controls (n = 2 per treatment group; Figure 1c & 1d).

**Figure 2:**
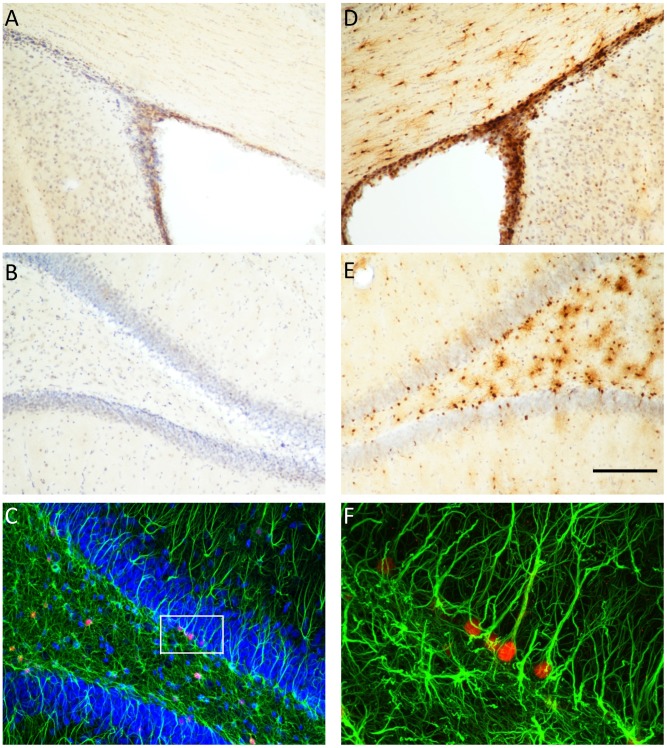
Thymidine kinase is expressed in the SVZ and DG, and co-localizes with GFAP positive cells. Thymidine kinase (TK) positive cells are detected in the subventricular zone (SVZ) and dentate gyrus (DG) of GFAP-TK rats, but not in wild type controls. Panels A and B show controls and panels D and E show GFAP-TK rats, Panel C shows double labeling of TK (red) and glial fibrillary acidic protein (GFAP, green) in the DG of a GFAP-TK rat. Higher magnification of a segment of panel C shows that TK positive (red) cells co-localize with GFAP staining. Scale bar represents 200 µm for panels A, B, D & E.

We used double-labelling immunohistochemistry to determine the specificity of TK expression in the brain of the GFAP-TK rat (Figure 2). TK expression was restricted to the hippocampus and the subventricular zone (SVZ). We did not observe any staining in neocortical regions. While many cells stained positive for GFAP but negative for TK, we found no cells that were GFAP negative and TK positive. Staining with a neural stem cell marker (Sox2 [44]) demonstrated that TK staining co-localized with Sox2 positive cells in the dentate gyrus, indicating that TK expression was restricted to neural stem cells as expected (Supplemental Figure S1). Thus at the single-cell level TK expression is restricted to GFAP-expressing cells in the hippocampus and SVZ.

### GFAP-TK rats exhibit reduced anxiety

The effect of neurogenesis ablation on anxiety was assessed using approach-avoidance conflict paradigms. After eight weeks of systemic GCV administration animals were administered a novelty-suppressed feeding test. This induced a high level of anxiety in the experimental cohort, with less than a quarter of the cohort consuming a novel food during the nine-minute experiment. GFAP-TK rats were significantly more likely to eat (8 out of 21, one rat untested) compared with wild type controls (1 out of 18, df = 1,37, P = 0.011, logistic regression), suggesting reduced anxiety in the transgenic group.

**Figure 3:**
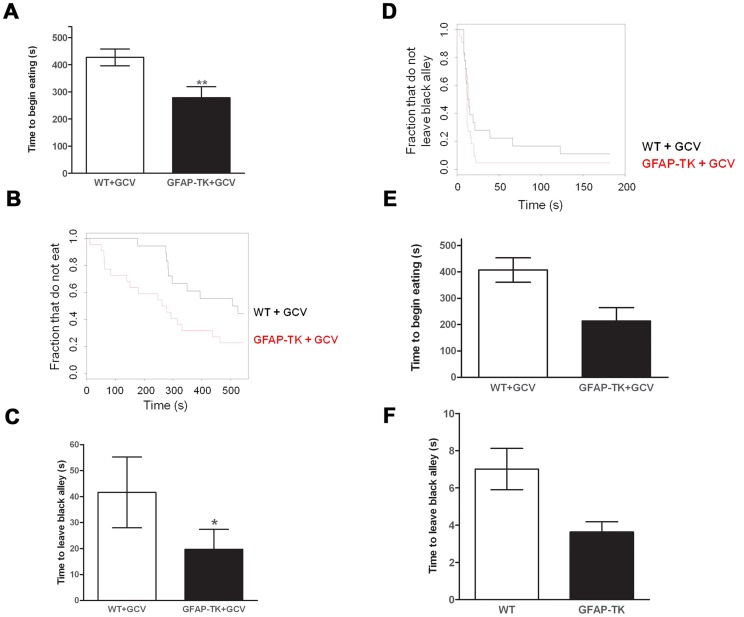
Ablating adult neurogenesis decreases anxiety. **A** Time to begin eating a novel food in a novel environment, expressed as the mean latency (± s.e.m). **B** Time to begin eating as a survival plot (black line = wild type, n = 18, Red line = GFAP-TK, n = 22). **C** Time to emerge from a black alley and enter a white alley expressed as the mean latency (± s.e.m). **D** Time to emerge from a black alley survival plot (black line = wild type, n = 18, Red line = GFAP-TK, n = 22). Grey vertical line at right side of survival plots represents the termination of the experiment. *p<0.05, **p<0.01 significantly different from GCV-treated wild type controls. **E** Time to begin eating a novel food and **F** Time to emerge from a black alley in untreated WT (n = 26) and GFAP-TK (n = 18) rats.

This effect was confirmed in a second, and less anxiogenic, novelty-suppressed feeding test, during which the GFAP-TK rats exhibited a significant decrease in latency to begin eating the novel food (Figure. 3 a & b, wild type n = 18, GFAP-TK n = 22, P = 0.0059; using a survival analysis that took into account both the time to make initial contact with the food and the effect of cohort). The GFAP-TK rats were still significantly more likely to eat compared to the WT controls (U = 133, z = −2.4, p<0.01; Mann Whitney-U test). Importantly, we found no effect of genotype on the time taken to make initial contact with the food (survival analysis, P = 0.13), suggesting that there was no difference between the two groups in terms of the ability to detect or attend to the food.

In a non-appetitively motivated black/white alley emergence test the GFAP-TK rats again exhibited decreased anxiety. This was shown by a significantly reduced latency to leave the black alley in which they were placed, and to enter into the white alley of the apparatus, compared with wild type controls (Figure. 3 c & d, wild type n = 18, GFAP-TK n = 22, P = 0.014, first taking into account the effect of cohort in a survival analysis). There was no effect of genotype on the number of crossings between the black and white alleys (two-tailed unpaired t(38) = 1.3, P = 0.21), suggesting that this effect was not due to general hyperactivity in the GFAP-TK rats.

At the end of the experiment we assessed neurogenesis in the cohort and confirmed almost complete ablation of newborn neurons. There was a 98.1% decrease in DCX-positive cells in the dentate gyrus of GFAP-TK rats compared to GCV-treated wild type controls (wild type: 6279.33±423.56, n = 18 vs. GFAP-TK: 119±37.36, n = 22; mean ± s.e.m, ANOVA, p = 4.75E-16).

We next considered whether the effects we observed might be due to the site of integration of the transgene. Therefore we compared transgenic animals, without GCV administration, to littermate, age matched controls. We tested the cohort in the same novelty-suppressed feeding and black/white alley tests. Using a survival analysis we found that the transgenic animals had reduced latency to eat the novel food, but this was not significant at the 5% level (26 GFAP-TK rats and 18 wild types, P = 0.056, Figure 3e and 3f). We also observed a trend towards reduced time to enter the white alley of the black-white alley emergence test, compared with wild type controls (P = 0.061). While these results do not exceed a 5% significance level, they also do not exclude the possibility that there is a difference between untreated transgenics and wild types, suggesting that the effect on anxiety could reflect integration of the transgene.

### Chronic ablation of adult neurogenesis has no effect on cued or contextual fear conditioning

Fear conditioning was elicited by pairing a neutral stimulus (a 100 dB tone, the conditioned stimulus (CS)) with an aversive stimulus (mild foot shock, the unconditioned stimulus (US)) in a distinctive context. Following CS-US pairings, presentation of either (i) the training context or (ii) the CS alone (presented in a different context) generates a fear response, assessed by measuring freezing behavior on subsequent days. After 8 weeks of systemic GCV administration rats underwent one day of conditioning trials. During the first 120 s of the conditioning session, prior to the delivery of any tones or shocks, baseline activity levels decreased and were similar across the two genotypes. A repeated measures ANOVA showed a main effect of time bin [wild type n = 7, GFAP-TK n = 10, F(5,75) = 3.4, p = 0.008], but no effect of genotype [F(1, 15) = 0.18, p = 0.68] or time x genotype interaction [F(5,75) = 0.79, P = 0.56].

**Figure 4:**
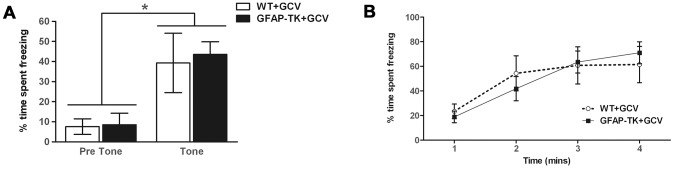
Ablating adult neurogenesis does not affect fear conditioning. **A** Freezing behavior of GCV-treated wild type (n = 7) and GFAP-TK (n = 10) rats during 60 s preceding a tone previously paired with a shock (Pre Tone) and during the first 20 s of tone presentation (Tone). Data represent % time spent freezing during each time period (± sem). **B** Freezing behavior of GCV-treated wild type (n = 7) and GFAP-TK (n = 10) rats in a context previously associated with shock presentation. Data represent % time freezing during 4 minutes (60 s time bins; ± sem). *p<0.05 tone significantly different from pre-tone. Wild type data are represented by an open circle connected by an interrupted line, GFAP-TK data are represented by filled squares and a solid line.

Ablation of adult neurogenesis did not significantly affect cue conditioning (Figure 4a). The tone was found to cause a significant increase in freezing behavior [F(1,15) = 26.38, p = 0.001], indicating that animals had been conditioned, but there was no significant effect of genotype [F(1,15) = 0.072, p = 0.79], or tone x genotype interaction [F(1,15) = 0.006, p = 0.80]. Contextual conditioning was also unaffected by a loss of adult neurogenesis, with both genotypes exhibiting equivalent levels of freezing to the context in which they had received a shock (Figure 4b). A repeated measures ANOVA showed a significant effect of time bin [F(3,45) = 18.94, p<0.0001], but no effect of genotype [F(1,15) = , p = 0.93] or time bin x genotype interaction [F(3,45) = 1.02, p = 0.39].

### Chronic ablation of adult neurogenesis has no effect on spatial reference memory in the water maze

**Figure 5:**
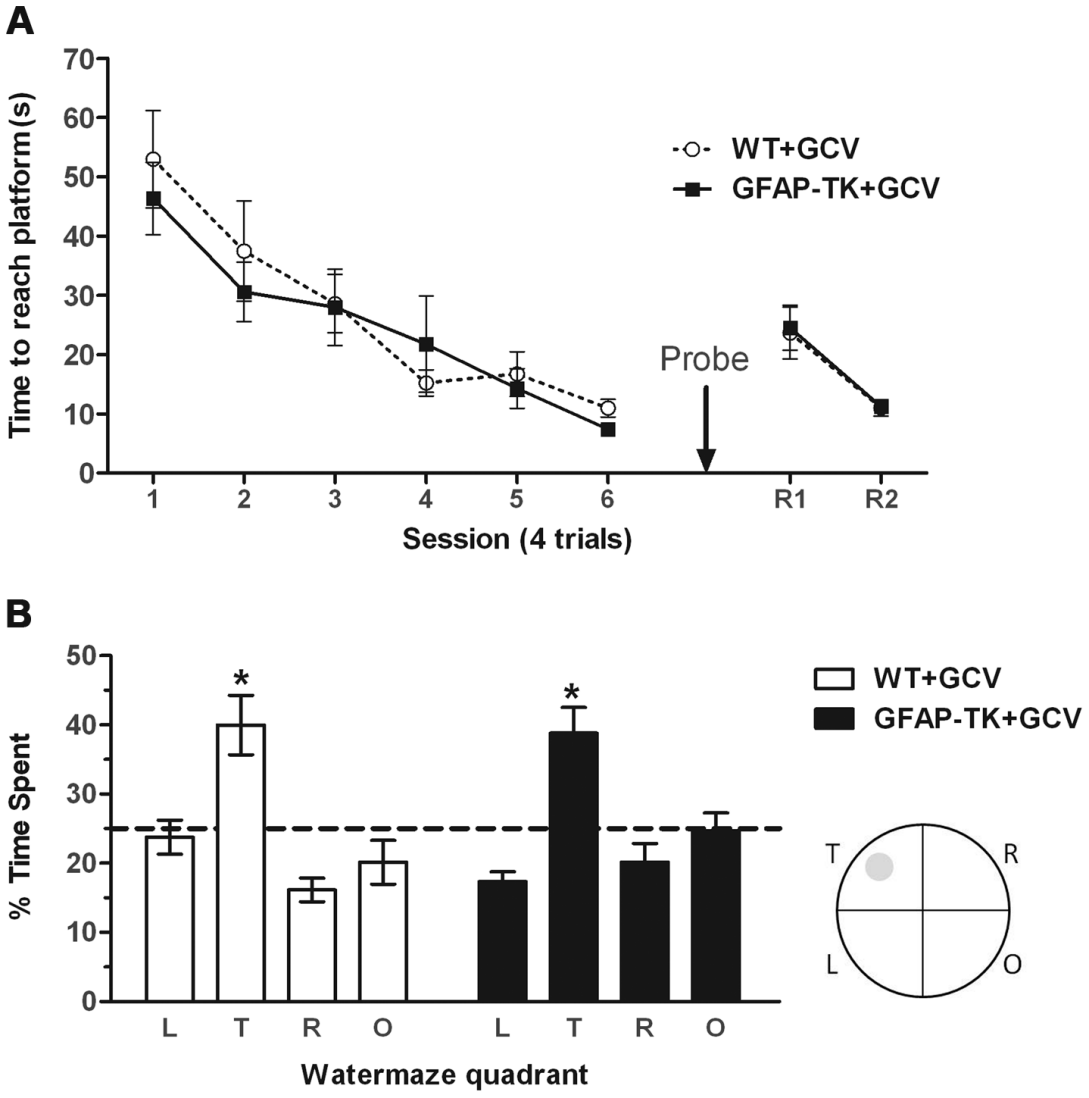
Ablating adult neurogenesis does not affect spatial reference memory in the water maze. **A** Time spent swimming to reach a submerged platform by GCV-treated wild type (n = 7) and GFAP-TK (n = 10) rats during the acquisition and reversal phase of a spatial reference memory water maze task. Each data point represents the mean score (±sem) per session (1 session consists of 4×90 second trials). R1/R2 represents a reversal phase, where the platform was moved to the opposite quadrant of the pool. **B** Percentage time spent swimming in each quadrant by GCV-treated wild type (n = 7) and GFAP-TK (n = 10) rats during the probe trial. L = adjacent left quadrant, T = target quadrant, R = adjacent right quadrant, O = opposite quadrant. Interrupted horizontal line represents a value of chance. Data represent mean % time (±sem) during a 60 s trial. *p<0.05 significantly different from time spent in other quadrants. Wild type data are represented by an open circle connected by an interrupted line, GFAP-TK data are represented by filled squares and a solid line.

Latencies and total distance swum to reach the platform decreased during the acquisition phase of the water maze task (significant effect of session on time spent swimming [F(5,75) = 17.42, p<0.0001; wild type n = 7, GFAP-TK n = 10] and distance swum [F(5,75) = 22.43, p<0.0001], suggesting that rats were able to learn the location of the platform and adjust their swimming behavior to a more efficient path accordingly (Figure 5a). However, there was no effect of genotype on swim latencies [F(1,15) = 0.35, p = 0.56] or distance swam [F(1,15) = 0.74, p = 0.40]. Furthermore, there was no significant interaction between genotype and session for either time spent swimming [F(5,75) = 0.50, p = 0.78] or distance swum [F(5,75) = 0.74, p = 0.59], suggesting that both groups learned the task at an equivalent rate.

The probe trial demonstrated that the rats could remember the position of the platform when it had been removed from the pool, as shown by significantly more swimming activity in the target quadrant compared to the other three quadrants (main effect of quadrant [F(2,45) = 16.56, P<0.0001]. A post-hoc pair-wise comparison showed that the time spent in the target quadrant was significantly different to all of the three other quadrants (p<0.05) for both genotypes (Figure 5b). However, a lack of genotype x quadrant interaction [F(2,45)1.17, p = 0.33] and an independent t-test showing there to be no difference between genotypes in the time spent in the target quadrant [t(15) = 0.20, p = 0.84], suggests that both GCV-treated wild type and GFAP-TK rats were able to remember the platform location to an equal extent. There was also no effect of genotype on total swim path length during the probe trial [t(15) = 0.53, p = 0.96].

The GFAP-TK rats were also unimpaired in their ability to learn a new platform location in a now familiar spatial environment (a form of spatial reversal in which the platform was moved to the opposite quadrant of the pool). A repeated measures ANOVA showed an effect of trial [F(7,105) = 14.70, P<0.0001], indicative of reversal learning, but a lack of any genotype effect [F(1,15) = 0.32, P = 0.58], or trial x genotype interaction [F(7,105) = 0.49, P = 0.84].

### Chronic ablation of adult neurogenesis has no effect on spatial working memory in the radial maze

The spatial working memory task in the radial maze involved training animals to enter arms of the 12-arm maze to retrieve food rewards. The rewards were not replenished during a trial, so the animal had to use spatial cues to remember which arms had already been visited and adjust their behavior accordingly. Re-entering a previously visited (now unrewarded) arm was recorded as an error and provides a measure of hippocampal-dependent spatial working memory [45], [46], [47].

**Figure 6:**
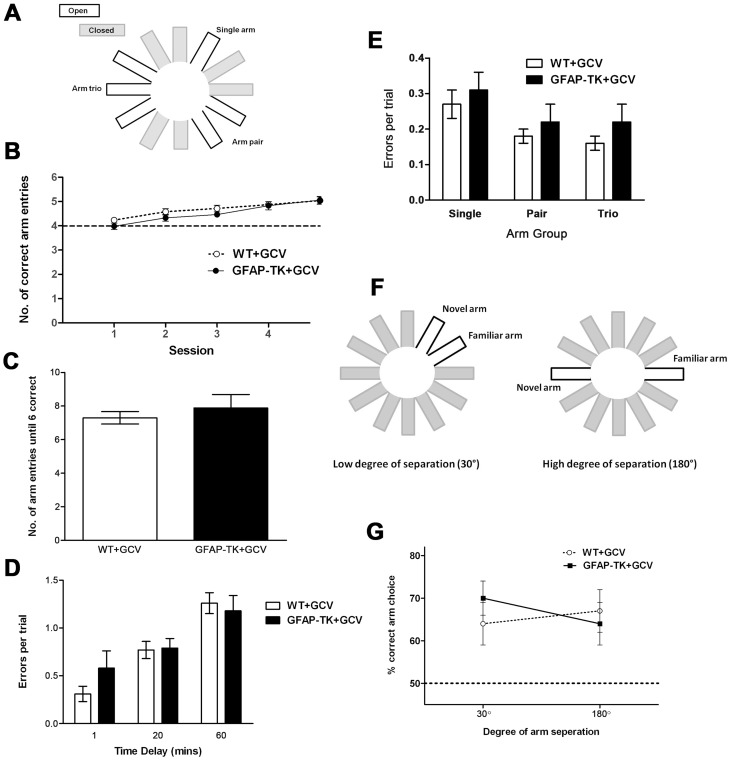
Ablating adult neurogenesis does not affect spatial working memory in the radial maze. **A** Arm configuration for radial maze task. **B** Mean number (± sem) of correct arm entries made during a maximum of 6 entries per trial, by GCV-treated wild type (n = 13) and GFAP-TK (n = 13) rats. **C** Number of arm entries made before all 6 arms had been visited in a trial, by GCV-treated wild type (n = 13) and GFAP-TK (n = 11) rats. The mean score (± sem) is shown for four sessions (4 trials per session). **D** Rats made 3 initial arm choices, followed by a 1, 20 or 60 minute delay, followed by 3 final arm choices. Data show the number of errors made during the final 3 arm choices, by GCV-treated WT (n = 13) and GFAP-TK (n = 11) rats. Each data point represents the mean score (± sem) for 3 trials for each rat. **E** Number of errors made per trial into the single arm, pair of arms and arm trio, by GCV-treated wild type (n = 13) and GFAP-TK (n = 11) rats. Data are adjusted according to the number of arms in each group (e.g. total number of arm entries into the trio was divided by 3). Each data point represents the mean score (± sem) per trial. **F** Arm configuration for the binary choice delayed non-matching to place radial maze task. **G** The percentage of trials (± sem) in which the novel arm was correctly chosen, by GCV-treated wild type (n = 11) and GFAP-TK (n = 9) rats in the delayed non-matching to place task. Wild type data are represented by an open circle connected by an interrupted line, GFAP-TK data are represented by filled squares and a solid line. The interrupted horizontal line represents chance levels of performance.

In our first experiment (phase one) we assessed spatial working memory by counting the number of correct arm entries made during the first six choices in each trial. A trial was terminated when six arm entries had been made, irrespective of the number of errors or rewards collected. Wild type and GFAP-TK rats were equally able to perform this radial maze spatial working memory task (Figure 6b). A repeated measures ANOVA showed a significant effect of session [F(4,96) = 19.56, P<0.0001; wild type n = 13; GFAP-TK n = 11] with rats making less working memory errors as phase one progressed, but no effect of genotype [F(1,24) = 1.68, p = 0.21], and no genotype x session interaction [F(4,96) = 0.66, p = 0.62]. We then gave the rats further training on the radial maze task, but now allowing them to remain on the maze until they had collected all 6 rewards (Figure 6c). There was still no effect of genotype on performance [F (1,22) = 0.52; p = 0.48].

We then asked whether new-born neurons might influence the ability to maintain a memory trace over time. We introduced a time delay (1, 20 or 60 minutes in a counterbalanced design) between the first three and last three arm choices of the radial maze protocol. The effect of the delay was assessed as the number of working memory errors made during the last three arm choices, as defined by a rat returning to a previously visited arm (Figure 6d). There was a significant effect of delay [F(2,44) = 25.41, p<0.0001], with a greater temporal delay leading to rats making more errors. There was however no effect of genotype [F(1,22) = 0.35, p = 0.56] or genotype x delay interaction [F(2,44) = 1.31, p = 0.28], suggesting that both genotypes were affected equally by each delay period.

### Chronic ablation of adult neurogenesis has no effect on pattern separation in the radial maze

While assessing working memory using the tasks described above, we were concurrently able to assess spatial pattern separation by varying the degree of spatial separation between the baited arms. We used a maze configuration with a single rewarded arm, a rewarded arm pair and a rewarded arm trio configuration, each separated by two closed arms. In accordance with the theory of pattern separation, the overlapping spatial cues used to identify each of the adjacent arms within the trio are predicted to generate greater interference, compared with the relatively unique spatial cues associated with the single arm. The high level of interference associated with the arm trio should require a greater amount of pattern separation to differentiate each arm from its adjacent neighbour, compared to the single arm where no, or very little, interference was present (Figure 6a). Differentiating the 3 arms of the trio should represent a greater load on granule cell processing and be associated with the highest number of working memory errors. In contrast, the single arm should be easily distinguishable and therefore associated with the least number of working memory errors.

When analysing the number of errors made in the various arm groupings (single, pair, trio; adjusted to account for varying number of arms), it was found that while there was an overall effect of arm group, performance was not affected by genotype (Figure 6e). A repeated measures ANOVA showed there to be a significant effect of arm group [F(2,44) = 15.16, p<0.0001], but with relatively more working memory errors being made in the single arm, than in one of the pair or trio of arms. However, there was again no effect of genotype [F(1,22) = 0.63, p = 0.44], and no genotype x arm grouping interaction [F(2,44) = 0.11, p = 0.90).

We also carried out a binary choice, delayed non-matching to place experiment. A naive cohort of GCV-treated wild type and GFAP-TK rats were tested on a radial maze in which the two arms of the choice phase were presented at either a high (180°) or low (30°) degree of spatial separation (analogous to that used by Clelland and colleagues [35]; Figure 6f). Neither the degree of arm separation nor the genotype of the rats had any effect on working memory performance (Figure 6g). A repeated measures ANOVA showed no overall effect of arm separation [F(1,18) = 0.14, P = 0.71; wild type n = 11, GFAP-TK n = 9], no main effect of genotype [F(1,18) = 0.08, P = 0.78], and no genotype by arm separation interaction [F(1,18) = 1.75, p = 0.20].

### Meta-analysis finds no significant effect of ablating adult neurogenesis but significant heterogeneity between studies

**Figure 7:**
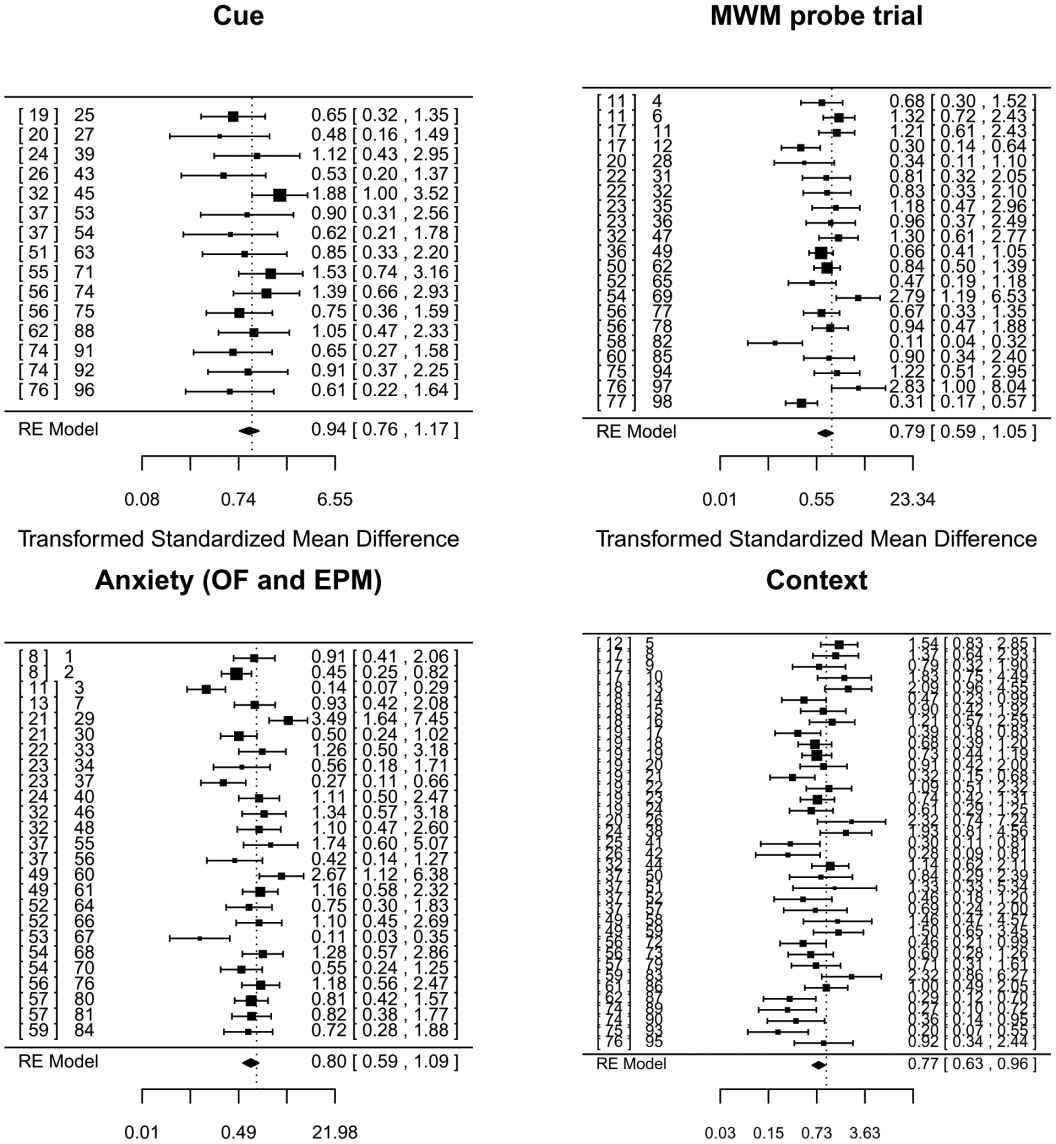
Meta-analysis of adult neurogenesis literature. Forest plot showing the results of studies examining the relationship between adult neurogenesis and three tests of learning and memory (contextual and cued fear conditioning, the probe trial of the Morris water maze (MWM)), and two tests of anxiety (total activity in the open-field arena (OF) and time spent in the open-arms of an elevated plus maze (EPM)). The figure shows the standardized mean difference for each study, the associated 95% confidence intervals and the pooled estimate, all based on a random-effects (RE) model and Hedge's estimator. Multiple entries for one publication arise when authors report analyses using different ablation methods (irradiation vs genetic for example) or variation in experimental protocols. On the right of each panel is the reference number for each publication, followed by a number that identifies the data set we extracted from the literature. This number refers to an entry in the supplemental table containing details of each data set and relevant covariates [8], [11], [12], [13], [17], [18], [19], [20], [21], [22], [23], [24], [25], [26], [32], [36], [37], [49], [50], [51], [52], [53], [54], [55], [56], [57], [58], [59], [60], [61], [62], [74], [75], [76], [77].

We carried out a quantitative analysis of the literature to see if conflicting results about the role of adult neurogenesis could be reconciled. Because of the diversity of tests used, we were able only to look at those measures with multiple independent reports. These are ethological measures of anxiety, fear conditioning (cue and contextual freezing) and spatial memory (probe trial of the Morris water maze). Open field activity and elevated plus maze tests were combined since they are jointly used to test anxiety. We identified 98 datasets suitable for analysis (Figure 7). [8], [11], [12], [13], [17], [18], [19], [20], [22], [23], [24], [25], [26], [32], [36], [37], [48], [49], [50], [51], [52], [53], [54], [55], [56], [57], [58], [59], [60], [61], [62]

Using a random effects model and Hedge's estimator, analysis of contextual freezing was significant at a 5% level (P = 0.043) but not significant at a significance threshold corrected for multiple testing (P = 0.05/4 = 0.0125). Results for the other measures were all non-significant at a 5% level (water maze, P = 0.14, cue conditioning P = 0.58, anxiety P = 0.15). Point estimates of effect size and confidence intervals are shown in the forest plot (Figure 7).

Heterogeneity tests for three analyses were significant (contextual freezing, Q(df 36) = 77.92, P<.0001, water maze, Q(df 20) = 54.6, P<.0001, and anxiety, Q(df 24) = 76.86, P<.0001), while those for the cue conditioning were not (Q(df 14) = 13.8, P = 0.46). The extent of heterogeneity is large: I^2^ for anxiety 71.1% (95% CI 59.7–84.9), for water maze 71.1% (95% CI 53.0–86.0) and 70.2% (95% CI 53.3–67.3) for context conditioning. The I^2^ statistic estimates (in percent) how much of the total variability in the effect size estimates is due to heterogeneity among the true effects. By contrast, I^2^ for cue conditioning is 0% with 95% confidence limits that do not include point estimates of the other measures (95% CI 0–52.7).

We attempted to identify sources of heterogeneity by examining the contribution of species, method and amount of knockdown, behavioral test used, and the length of time between training and the extinction test. However no moderator was significant (P-values>0.1) and at most accounted for 10% of the estimated amount of residual heterogeneity. This suggests other moderators, not considered in the model, are important.

## Discussion

We report a novel model for ablating adult neurogenesis in the rat. The chronic dosing of GCV to the GFAP-TK rat resulted in a reduction of DCX-positive cells by more than 98%, demonstrating this to be an effective approach for the inducible ablation of adult neurogenesis in rats as well as mice. To our knowledge, this is the first ‘pharmacogenetic’ rat model for reducing adult neurogenesis, and when compared to previously used methods, such as x-ray irradiation and antimitotic agents, the GFAP-TK rat demonstrates equal or greater efficacy. Using this model, we showed that ablation of adult neurogenesis affects neither spatial reference memory acquisition in a water maze, nor spatial working memory performance on a radial maze. There was also no effect on fear conditioning, in terms of either cue or contextual freezing. The GCV treated GFAP-TK rats exhibited reduced anxiety on two novelty-suppressed feeding tests and a black/white alley emergence test. However, we found that untreated GFAP-TK rats were also less anxious than littermate wild type controls. Although this result was not significant at the 5% threshold, it suggests that the emotional effect we have observed may be due, at least in part, to the site of integration of the transgene.

While we have demonstrated that the GFAP-TK construct is an effective method to ablate adult neurogenesis in the hippocampus, achieving knock-down of 98%, it should be noted that the model does not only impair neurogenesis. GFAP is a marker of radial glial-like precursors, cells reported to act as multipotent and self-renewing neural stem cells. Consequently knock-down will affect not only neurons but glia [63]. Delivery of GCV is known to destroy dividing astrocytes in the GFAP-TK mouse [64]. Thus any effects found with our model cannot wholly be attributed to a failure to produce new neurons. However it is important to point out that this additional effect on glia does not impact on the observation that preventing adult neurogenesis does not affect spatial performance or contextual fear conditioning in our study.

Our most striking finding is that we could find no significant difference in pattern separation abilities between GCV-treated controls and transgenic rats. Rats with no adult neurogenesis did not differ from wild type rats in their ability to differentiate arms with varying levels of spatial interference (Figure 6e). In a second task, a binary choice, delayed non-matching to place task, transgenic rats again performed as well as wild type controls at arm configurations of both low and high spatial separation (Figure 6g). The design of the second task was directly based on a previous study that successfully found an effect of ablating adult neurogenesis on spatial pattern separation in mice [35].

One possible explanation is that the configuration of spatial cues in our radial maze experiments were not sufficient to require spatial pattern separation and thus to reveal any group differences. In this respect, it is first important to point out that our spatial radial maze tasks are exquisitely sensitive to hippocampal manipulations, including hippocampal lesions [65].Furthermore, it should be noted that in our first radial maze study, rats actually made more working memory errors into the single rewarded arm than into the trio of adjacent arms (allowing for the total numbers of arms within a group). Subsequently, in our second radial maze experiment we found no difference in working memory performance levels on trials when the arms presented on the choice run were separated by either 30 or 180 degrees. Thus, our control rats were not finding this task any harder when they were required to choose between arms that were closer together. It is important to point out that this was also true for the previously cited radial maze study [35]. The authors observed that the choices made by their control animals were, numerically at least, more accurate on trials when the arms that had to be discriminated were closer together.

A more general question therefore remains as to the precise role that the hippocampus plays during pattern separation on the radial maze. Although a role for the hippocampus in disambiguating between similar or overlapping inputs seems highly likely, the source of this ambiguity in behavioral tasks like the radial maze is often poorly defined. Recently we have shown that the ambiguity on the radial maze task may arise, not from the overlap of the extramaze spatial cues between one arm of the maze and the next, but rather from the similarity of the intramaze cues that are common to all of the arms of the maze (e.g. all of the arms have the same doors, floors, side walls, food wells [65]). Thus, although the hippocampus may indeed play an important role in pattern separation for disambiguating between the arms of the radial maze, this does not reflect the ability to distinguish between overlapping extramaze spatial cues. Rather it reflects the ability to use the extramaze spatial cues as conditional cues, to indicate whether the local intramaze arm cues, that are common to all of the arms, are associated with either reward or non-reward. Nevertheless, whatever the nature of the underlying requirement for pattern separation on a spatial radial maze task, the present data suggest that adult hippocampal neurogenesis may not always be essential for this process.

A major aim of this study was to strengthen a consensus on the function of adult neurogenesis, but our findings have, if anything, further added to the confusion in an already conflicted literature. While there is acknowledged discrepancy in the findings for memory and emotional processing [66], [67], [68], to date the findings for pattern separation have been robust: six studies have reported that pattern separation is altered when adult neurogenesis is altered [35], [36], [37], [38], [39], [40].

One possibility is that the effect is present, but we were unable to detect it. We think this is unlikely because we did not observe a trend in the expected direction of the effect, suggesting that lack of power cannot account for our findings. For example, we found that the transgenic animals performed numerically better than controls (although not significantly) on the more demanding separation task (maze arms separated by 30 degrees, Figure 6f and 6g), and this difference lessened on the purportedly easier task of distinguishing between two arms separated by 180 degrees.

What might account for the discrepancy? Here the findings of our meta-analysis (Figure 7) are relevant in two ways. First, if there is an effect of adult neurogenesis on any of the phenotypes measured, then that effect is relatively small, since we could not detect a clear signal in the combined studies. One possibility that we cannot exclude is that there simply is no true effect; the current literature represents a pattern of results expected from numerous underpowered studies. Second, the meta-analysis reveals remarkable heterogeneity among the studies and this suggests an alternative explanation for the current confusion. While we could not fully attribute heterogeneity to a number of likely sources (variation in species, method of knockdown, behavioral test used, or the length of time between training and the extinction test), we did find that the extent of heterogeneity varies significantly between the phenotypes; it is less, and significantly less, in the one task that is for the most part hippocampal independent: cue conditioning [69]. One possible explanation for heterogeneity is variation in stress levels which could interact with hippocampal function [66], [68].

Recently a number of commentators attempting to synthesize the conflicting findings for adult neurogenesis have pointed to the importance of environmental regulation [39], [66], [68]. Sahay and colleagues (2011) proposed that changing levels of neurogenesis in the dentate gyrus represents a long-term adaptive response to different environments by shifting the balance between pattern separation and pattern completion [39]. Dranovsky and Leonardo argue that young neurons assign stress salience to novelty during learning [68] while Glasper and colleagues argue that the impact of immature neurons may depend on whether they are born in conditions of low or high stress: neurons born in conditions of low stress facilitate exploration and learning; under high stress conditions, animals lack stress-induced GABAergic inhibition of granule cells leading to more anxiety-like behavior [66].

The relationship between stress and/or glucocorticoid levels and hippocampus-dependent behaviors follows an inverted U shaped function, such that any intervention that interacts with the stress axis could have opposing effects relative to control subjects, depending on the background levels of stress and/or arousal. Deviations in either direction from a state of medium arousal (which is the peak of the inverted U), will lead to performance decline. While increases in stress/arousal at the lower end of the curve will increase performance, the same is achieved by decreasing stress/arousal at the upper end. Snyder and colleagues report that mice lacking adult hippocampal neurogenesis displayed altered glucocorticoid responses to stress [9]. These neurogenesis-deficient mice also exhibited increased levels of anxiety on the ventral hippocampus dependent novelty suppressed feeding test, but, critically, only after exposure to an acute stressor.

To conclude, adult born neurons in the hippocampus may produce subtle changes in behavior in response to long-term changes in the animal's environment or current life situation. Integrating recent data on the interaction between the stress axis and adult hippocampal neurogenesis may be important if we are fully to understand the role of these new-born neurons.

## Materials and Methods

### Ethics statement

All animal work was conducted according to UK guidelines and approved by the UK Home Office.

### Generating the GFAP-TK rat

The GFAP-TK rat was generated by pronuclear injection of a ‘*Gfap-Tk*’ Bacterial Artificial Chromosome (BAC) construct, engineered using ‘RedET’-mediated homologous recombination methods (GeneBridges, Germany). A gene encoding the Herpes simplex virus thymidine kinase enzyme (HSV-TK, referred to hereafter as Tk) was inserted into a plasmid vector with an R6K origin of replication, upstream of a Simian Virus-40 (SV40) polyadenylation (pA) sequence (gift from Francis Stewart, TUD, Germany). A Flp Recombinase Target (FRT)-flanked neomycin antibiotic resistance gene (FRTneo^r^FRT, Gene Bridges, Germany) was inserted downstream of the *Tk*-pA to facilitate targeting into a *Gfap-*containing BAC. The *Tk*-pA-FRTneo^r^FRT construct was inserted into a BAC (CH230-M13) to replace the start codon of the full rat *Gfap* gene (Figure 1a). The BAC CH230-380M13 (CHORI, CA, USA) was 178 106 base pair (bp) in size, contained the full sequence of the rat *Gfap* gene (8 674 bp) with an additional 154 271 bp of 5′, and 15 161 bp of 3′ sequence. It has been shown that a *Gfap* promoter cassette containing only 2.2 kb of 5′ sequence and 1.4 kb of 3′ sequence is sufficient to drive endogenous GFAP-like expression of LacZ in mice [42]. The neo^r^ gene was removed from the BAC *in vitro* by FLP-mediated recombination.

The modified BAC was injected into the pronucleus of 326 fertilized Sprague Dawley oocytes, which were transferred into 12 pseudopregnant foster mothers [70]. Genomic DNA was extracted from the tails of 101 pups (Nucleon Genomic DNA Extraction Kit, Tepnel Life Sciences, Manchester, UK) and analysed by PCR using primers optimised for *Tk* detection. Genotyping identified 6 founder rats (3 male) positive for insertion of the *Gfap-Tk* transgene. The testes of the male rats were reported to be aspermatic, a known side effect of expressing HSV-TK in rodents [71]. This infertility restricted breeding to heterozygous GFAP-TK females and wild type males. Only one of the three transgenic females was found to generate transgenic pups, leading to the assumption that this was the only fertile founder where the transgene had integrated into the germline.

To ensure that the presence of the transgene alone did not affect adult neurogenesis, doublecortin (DCX)-positive cells were counted in the dentate gyrus of non-treated 7-week old wild type and GFAP-TK rats. Counts were made in 4 coronal brain slices per rat, taken from a 1 in 12 series (starting at −2.5 mm Bregma). To investigate the efficacy of the GFAP-TK rat model and determine the minimal effective dose of ganciclovir (GCV), 8-week old male GFAP-TK rats were implanted with a single subcutaneous slow-release osmotic minipump (2ML4, Alzet, CA, USA) for 4 weeks, containing either vehicle or GCV at a dose of 2, 4, 8 or 10 mg/kg/day.

#### Histology

Rats were deeply anaesthetised with pentobarbitone (300 mg/kg, i.p) and perfused transcardially with 4°C 0.9% saline followed by 4% paraformaldehyde (PFA) in phosphate buffered saline (PBS). Brains were removed and allowed to post-fix for 24 hours in 4% PFA. Brains were cryoprotected by immersion in PBS with 30% sucrose until equilibrated. Brains were cut in a coronal plane at a thickness of 40 µm using a freezing microtome, and preserved as free-floating sections in an antifreeze solution stored at −20°C. Free-floating sections were washed in PBS (3×5 minutes) to remove antifreeze and then immersed in 3% H_2_O_2_ for 10 minutes. Brains were rinsed in PBS (3×5 minutes) and incubated with goat anti-DCX C-18 primary antibody (1∶200 in 3% Triton-X with normal horse serum, Santa Cruz Laboratories) overnight at room temperature. The next day, sections were washed in PBS (3×5 minutes) and incubated in biotinylated anti-goat secondary antibody (Vectastain ABC elite kit, goat IgG, Vector Laboratories, USA) according to the manufacturer's instructions. Sections were rinsed in PBS (3×5 minutes) and left to react for 10 minutes with a DAB tablet (Sigma). Sections were slide mounted and dried and then immersed in Cresyl Violet (0.5%) for a few seconds (depending on stain strength). Slides were washed in water and then dehydrated through a series of increasing ethanol concentrations (75%, 80%, 95%, 100%), and finally immersed in Histoclear for 1 hour.

For the GFAP and TK double labelling fluorescent immunohistochemistry, staining was performed on 40 µm floating sections. The sections were incubated in anti-GFAP antibodies (Sigma; 1∶1000) and anti-HSV-TK (a gift from Michael Sofroniew) 1∶1000) overnight. Following three washes in PBS (5 min each), sections were incubated with the fluorescent secondary antibody (1∶200, Alex Fluor 488 and Alex Fluor 568, Invitrogen) for 2 h in 0.3% Triton/PBS with 2% of goat serum. Images of sections were captured on a Zeiss LSM 510 META confocal microscope.

### Effects of ablating adult hippocampal neurogenesis on anxiety

#### Animals

Rats were group-housed in standard open top cages with bedding and a cardboard tube for enrichment. Food and water were available *ad libitum*, except where rats were food deprived for appetitively-motivated tests. Lighting was kept on a 12 hour cycle with lights coming on at 7am. Assessment of GCV-treated GFAP-TK rat body weight at the end of experiment 1 showed there to be no significant difference compared to drug-treated wild type littermate controls (wild type = 429±15.10 g, GFAP-TK = 395±20.47 g) [t(15) = 1.23, p = 0.24].

#### GCV preparation and dosing regime

Rats were implanted with a GCV-containing osmotic minipumps at 8 weeks of age. This pump was replaced after 4 weeks. Two weeks after pump replacement (i.e. 6 weeks after the start of GCV dosing) behavioral testing commenced and continued for 2 weeks until the rats were perfused at 16 weeks of age. Brains were processed for DCX immunohistochemistry, as described above, to verify genotype and quantify neurogenesis. GCV was prepared, based on minipump delivery rate and rat weight, to deliver an approximate dose of 12 mg/kg/day at the point of pump implantation, decreasing to 10 mg/kg/day (due to an increase in rat weight) by the time the pump was exhausted 4 weeks later.

#### Unconditioned tests of anxiety

To investigate the effects of reducing adult hippocampal neurogenesis on emotionality, we chose unconditioned, ethological anxiety tests that we have shown to be reliably and reproducibly sensitive to hippocampal (particularly ventral hippocampal) lesions. In previous studies we have shown that hippocampal lesions resemble anxiolytic drugs in reducing anxiety on these very same tasks.

#### Food neophobia 1

Food neophobia tests generate anxiety via the conflict produced by presenting a calorific, but novel, food reward to a hungry animal in a novel environment. The apparatus for the first anxiety test consisted of a standard wooden T-maze raised to a height of 76 cm above floor level but with no side-walls. An insurmountable block of wood was placed half way along one arm to create a rectangular platform of 50×9 cm at the end of the arm. A food well was positioned at the end of this platform. Food was removed from home cages the night before testing to increase motivation. Each experimental rat was removed from its home cage approximately 10 minutes prior to testing and kept in a novel transport cage in a novel holding room to equilibrate arousal levels.

At the start of the experiment a single sucrose pellet (Noyes pellet, Sandown Scientific, UK) was placed in the food well. The rat was placed facing the wooden block (away from the reward) and the latencies (i) to make contact with the pellet and (ii) begin eating the pellet during a 180 second (s) trial were recorded. If the rat had not eaten within 180 s, it was removed from the apparatus and returned to its home cage. After approximately 10 minutes (no less than 6 minutes, no longer than 12 minutes) the rat was placed back on the apparatus for another 180 s. This was repeated until the rat had either eaten the pellet or spent a total of 9 minutes on the maze, in which case a latency score of 540 s was recorded. The maze and food well were cleaned with a 10% ethanol solution between trials.

#### Food neophobia 2

The second food neophobia test involved a similar protocol to that above, but used different apparatus, in a novel room, and used a novel food reward. Rats were placed on a 38 cm diameter circular disc of 3 mm thick translucent red Perspex. This was fixed in the centre to a supporting pole at a height of 77 cm above floor level. The platform was unsteady under the weight of the rat. A piece of sweetcorn (Green Giant Original Niblets, General Mills, UK) was placed in a food well in the centre of the platform. At the start of the test, a rat was placed tangentially to one side of the food well so that it registered the presence of the reward, but was not directly facing it. Latency to begin eating during a three-minute trial was recorded. Animals that did not eat were removed from the maze and returned to their home cages. Animals were re-tested, with a maximum of 3 trials (each of maximum duration 180 s) as described for the food neophobia test 1. Food neophobia tests 1 and 2 were separated by a period of two days.

#### Black/white alley

The black/white alley had a floor dimension of 9×122 cm and was enclosed by 30 cm high walls. The black alley and white alley were joined to form a single long lane. Experimental rats were placed in the black alley facing, and approximately 5 cm away from, the end wall. The latency to cross into the white alley (all four paws out of the black alley) was recorded for each rat during a single three-minute trial. If the rat did not leave the black alley after this time, the trial was terminated and a score of 180 s was recorded. The alley was cleaned with a 10% ethanol solution between rats. Latency to reach the end of the white alley, time spent in each alley and number of transitions between the alleys, were also recorded.

### Effects of ablating adult hippocampal neurogenesis on spatial reference memory

#### Water maze spatial reference memory task

The water maze was a 2 m diameter circular white pool with 60 cm high walls. The pool was situated in a novel room that was evenly lit and contained prominent extra-maze cues. The pool was filled with water (25±1°C), with milk added to aid tracking of the rat and obscure the position of the platform. In order to escape from the water, rats were required to locate and climb onto a hidden escape platform (10 cm diameter), submerged approximately 1 cm below the water surface. The position of the platform remained in a fixed position for each rat throughout the acquisition phase of the test. The platform was located in the centre of either the NW or SE quadrants, (counterbalanced with respect to genotype) and positioned 50 cm from the closest point of the wall of the pool.

Rats were trained to locate the escape platform over 3 days. Rats completed two sessions per day, one in the morning and one in the afternoon, with each session consisting of 4 trials. For each trial, a rat was placed in the pool approximately 5 cm from, and facing, the side of the pool at one of 8 start locations (N, NE, E, SE, S, SW, W, NW, pseudo-randomly allocated across trials). Rats were allowed to swim until they had found the platform or for a maximum of 90 s, at which point they were guided to the platform by the experimenter. An inter-trial interval of 45 s was used, of which 30 s was spent sitting on the platform to allow the rat to rest and orientate itself within the room. The time to reach the platform and the swim path length were recorded.

On day 4, a probe trial was carried out to assess how well the rats had learned the spatial location of the platform. The platform was removed from the pool and the rats were allowed to swim freely for 60 s. The time spent in the training quadrant, relative to the other three quadrants, was used as a measure of spatial learning. Because time spent in a quadrant was not independent from the time spent in the remaining 3 quadrants, *p*-values were adjusted to reflect the reduced degrees of freedom with respect to both the main effect of quadrant and group x quadrant interaction.

On day 5, a test of spatial reversal was carried out. A morning and afternoon session were conducted, as in the acquisition phase, but with the platform moved to the centre of the opposite quadrant (e.g. NW moved to SE). Time to reach the platform and swim path length were recorded, with a maximum time of 90 s allowed for each rat.

### Effects of ablating adult hippocampal neurogenesis on fear conditioning

Animals were tested over three days using two distinct chambers. Chamber 1 (28 cm wide, 21 cm high, 22 cm deep; Med Associates Inc., IN, USA) had a roof and two walls made from Plexiglas, and 2 walls made from aluminium. The floor consisted of 18 stainless steel rods (0.4 cm diameter) that were connected to a shock generator. The chamber was housed within a sound attenuated and lightproof wooden casing (40 cm wide×35 cm high×40 cm deep). Chamber 2 was made entirely from Perspex (22 cm wide×25 cm high×20 cm deep), had an open top, and was housed within a different sound attenuated and lightproof box (40 cm wide×38 cm high×40 cm deep). Chamber 2 was further distinguished from chamber 1 by covering the floor with a black paper napkin, having the houselights off, and having a distinct odour (Sandalwood Essential oil, Body Shop Ltd, UK). The chambers were cleaned with a 10% ethanol solution between animals. In chamber 1 a house light, speaker and shock generator were interfaced with a personal computer and controlled by MED-PC V.4 software (Med Associates Inc.). In both chambers a video camera, connected to a PC, was mounted on the top of the chamber to record rat movement.

On the first day all rats were subjected to a 740 s conditioning protocol in chamber 1. During this period rats received five tone-shock pairings. Each 100 dB tone lasted 20 s and co-terminated with a 1 s 0.5 mA footshock (variable intertrial interval with a mean of 60 s). The first tone was presented 40 s after the rat was placed in the chamber. Rats were subjected to either a cue or context trial on day 2 and 3, the order of which was counter balanced across genotype.

To assess cue conditioning animals were placed in chamber 2. After 120 s the rats were presented with the same tone as that presented in the conditioning session. No foot shocks were given. Activity and freezing behavior were measured throughout the session. To assess contextual freezing animals were returned to chamber 1 for a period of 240 s. Activity and freezing behavior were measured throughout this session.

Activity and freezing behavior were assessed using ‘NIH image’ software (NIH, MD, USA). Activity and freezing in response to a cue were calculated as the mean number of pixel changes per second and % of time spent freezing, respectively, during the 60 s preceding the first tone (pre-tone), and during the 20 s presentation of this tone (tone). Activity and freezing in response to the context were calculated as the mean number of pixel changes per second and % of time spent freezing, respectively. Data were collated into 4 consecutive 60 s time bins.

### Effects of ablating adult hippocampal neurogenesis on spatial memory and pattern separation in the radial maze

#### GCV preparation and dosing regime

GFAP-TK rats and wild type littermate controls were implanted with a GCV-containing osmotic minipump at 8 weeks of age, which was replaced after 4 weeks, and again 4 weeks later. Behavioral testing was started 6 weeks following the first pump implantation and continued for 2 weeks until the third pump was inserted. After a week of recovery, behavioral testing continued for a further 3 weeks until rats were perfused and brains removed for DCX immunohistochemistry.

#### Radial maze apparatus

The radial maze consisted of a central arena comprising a 47 cm diameter dodecagon made from transparent Perspex, with 40 cm high transparent Perspex walls. Twelve equally spaced aluminium arms (60 cm by 10 cm) radiated out from this central arena, and were enclosed by low 3.5 cm high aluminium walls.

Rats were given access to 6 of the 12 arms. All 6 arms were rewarded at the start of a trial. The 6 rewarded arms were arranged such that there was a single rewarded arm, an adjacent pair of rewarded arms, and a cluster of 3 neighbouring rewarded arms. These ‘single arm’, ‘2-arm pair’ and ‘3-arm trio’ groupings were spatially separated by 2 ‘closed/unrewarded’ arms that the rat could never enter (Figure. 6a). Therefore, when a rat was exposed to this configuration, the three arm groups had the same level of ‘inter-group’ spatial interference (i.e. the spatial separation between each grouping), but had varying levels of ‘inter-arm’ spatial interference within each group (minimal interference for the single arm, maximal interference for the arm trio). The position of the 6 baited arms was counterbalanced across rats with respect to genotype and remained the same for each animal throughout the experiment. To habituate the rats to the apparatus, they were placed on the maze with their cage mates (2, 3 or 4 animals) for approximately 5 minutes, twice a day for 2 days, and were allowed to explore the entire maze. All doors were open and all arms remained rewarded at all times. This was carried out during the week prior to the start of testing (week 6 of GCV dosing).

#### Spatial working memory (phase one)

Phase one was carried out during the first 2 weeks of behavioral testing. Individual rats were placed in the central area and allowed to orientate themselves. After 10 s the trial was initiated by the experimenter concurrently opening 6 doors, according to the configuration described above, allowing the rat access to the 6 baited arms. When the rat had entered an arm (determined by all 4 paws leaving the central arena) all doors were lowered. When the rat had consumed the reward pellet (Noyes, Sandown Scientific, UK) the door was opened and the rat was allowed to return to the central platform. The rat was then confined to the central platform for 10 s to allow reorientation and prevent thigmotaxis- or proximity-influenced choice behavior before the next choice was initiated by all 6 doors being raised. The trial was terminated when 6 arm entries had been made, irrespective of the number of errors or rewards collected. Working memory was assessed by counting the number of correct arm entries (entries into previously unvisited arms) made during the trial.

#### Spatial working memory (phase two)

Phase two was carried out during weeks 4 and 5 of behavioral testing. The protocol used was identical to that described for phase one, but with the difference that the session was only terminated when the rat had made 6 correct arm entries (i.e. the rat had visited all 6 baited arms), thus providing further training on the task prior to the introduction of delays (see below). Working memory was assessed during phase two by counting the number of total arm entries made until all 6 rewarded arms were visited.

#### Effect of time delay

Phase three was carried out during week 6 of behavioral testing and involved increasing the memory load by introducing a delay half-way through the trial. Following the first 3 arm entries, the rats were removed from the maze and placed individually in a novel cage within the test room for a period of 1, 20 or 60 minutes. During this delay period the maze was rotated by 60 degrees anticlockwise and cleaned with 10% alcohol to limit the use of intra-maze cues in the subsequent memory retention phase. After the delay, the rat was returned to the maze and allowed to make the final 3 arm entries. The effect of a temporal delay on working memory was assessed by counting the number of errors (entries into previously visited arms) made during the final 3 arm choices.

#### Effect of spatial interference

The effect of spatial interference was assessed across all three phases of this experiment. The numbers of working memory errors (defined as an entry into a previously visited arm) made into each arm grouping (single, pair or trio), were calculated to assess the effect of spatial interference from adjacent arms. The data were analysed as the number of errors made per trial in the single arm, the arm pair, and the arm trio. The data from the arm pair and the trio were adjusted to account for the number of arms in each group (i.e. the total number of errors made were divided by 2 for the pair and 3 for the trio).

#### Delayed non-matching to place

We further investigated the role of adult born neurons in spatial pattern separation using a naive cohort of rats and a 2-arm delayed non-matching to place paradigm. Testing was carried out during weeks 9–12 of GCV dosing. Individual rats were placed on the central platform of the maze and allowed to orientate themselves. After 10 s the sample phase was initiated by the experimenter opening a single door and allowing the rat to access an arm of the maze. When the rat had consumed the reward pellet the door was opened and the rat was allowed to return to the central arena. The rat was then removed from the maze and placed in a transparent transport cage in the testing room for a period of 30 seconds. During this time the maze was rotated 60 degrees anticlockwise and cleaned with 10% alcohol to limit the use of intra-maze cues in the subsequent sample phase. The rat was then put back in the centre of the maze for ten seconds before the experimenter opened the doors to both the original arm (termed ‘familiar’ with regards to the allocentric, extra-maze spatial cues), and a ‘novel’ arm.

The novel and familiar arm were presented with either a low degree of spatial separation (30 degrees between arms, i.e. adjacent arms) or with a high degree of spatial separation (180 degrees between arms, i.e. opposite arms; Figure. 6e). The rat was rewarded for choosing a previously unvisited (relatively more novel) arm. If the rat re-entered the ‘familiar’ (now unrewarded) arm, this was recorded as a working memory error. After the rat had made a choice it was removed from the apparatus and returned to its home cage. The rats received one ‘low spatial separation’ and one ‘high spatial separation’ trial per day for 12 consecutive days, with the order of high vs. low trials counterbalanced across days. For the low separation trials the clockwise/anticlockwise relationship between the novel and familiar arms was counterbalanced across days for each animal. In order to reduce any effect of spatial interference between trials, the rats were not exposed to the same arm locations more than once in a day. Furthermore, the position of the novel and familiar arms was balanced across the 12 days to ensure that the rats were equally exposed to all of the 12 possible arm positions.

### Meta-analysis

Articles were identified using PubMed using the search terms “neurogenesis”, “adult”, and “behavior”. Once articles had been collected, bibliographies were then hand-searched for additional references. We included only articles that reported an ablation of neurogenesis, thus excluding those that used running or an enriched environment to increase neurogenesis. We included measures of fear conditioning (contextual and cued), elevated plus maze and open-field arena and measures of spatial reference memory since these were most commonly measured (for example there were too few reports using the Barnes Maze).

There are multiple measures that can be taken from each of the behavioral tests. We selected those that are most frequently reported and widely acknowledged as indices of the relevant psychological variable. From the open-field we used the total distance travelled, for the elevated plus maze the time spent in the open arms. We used the probe trial of the Morris water maze and the percent time spent freezing from the fear conditioning tasks. In some instances the same test was performed on separate cohorts of animals (for example where a group used both irradiation and genetic method to ablate neurogenesis). We treated these as independent samples for the meta-analysis.

From each article we extracted: 1) author(s) and year of publication; 2) methods (extent of neurogenesis knockdown, method of knockdown); 3) species (rat, mouse); 4) data (number of animals in test and control groups, mean and standard deviation (derived from the reported standard error of the mean where appropriate).

We dealt with heterogeneity in the literature by including all tests and methods of knockdown as separate experiments (i.e. we aimed for inclusiveness and never averaged results when papers employed multiple different study designs). Thus when tests are reported under different conditions (for example some papers use multiple methods for neurogenesis knock down) we included each result separately. Our inclusive strategy of data collation means that a single paper can have multiple entries in the same table. For example Deng et al tested contextual memory one day after training, in two cohorts of animals, the second of which began training mice 5 weeks after ganciclovir (GCV) was injected intraperitoneally [19]. Where the samples were separate (i.e. the same animals were not repeatedly tested) we treated this as a separate entry into the meta-analysis. Where the same data are reported in multiple publications, we kept just one instance (e.g. “the 6 weeks group only were previously published in Drew et al., 2010” [58]).

Many studies report tests carried out at multiple different time intervals, investigating the hypothesis that the effects of neurogenesis are delayed, so for example would only be apparent when animals are tested one month after training. Again we dealt with this multiplicity by including all results as separate entries into the meta-analysis. However when we tested whether variation in the time of testing contributed to heterogeneity in results we classified time of testing into two groups: early (when animals are tested within a week of training) and late (when animals are tested more than a week after training). In a small number of cases investigators repeatedly test the same cohort of animals (for example Deng et al administer a probe trial (of the Morris water maze) from the 4^th^ day of training and at 1, 2 and 3 weeks post training [19]). In these cases we take a single measure for the early time point (1 day after testing) and a single measure for the late time point (one month after training, or the nearest time point to one month), since these two times are those most commonly reported to evaluate early and long-term memory retention respectively. Supplemental Table 1 provides a summary of the experimental groups we extracted, the papers from which we extracted the data and a list of the covariates included in the meta-analysis).

Meta-analysis was carried out using the metaphor package in the R statistical language [72]. Due to the large amount of heterogeneity, data were analysed in a random effects framework.

## Supporting Information

Figure S1 Thymidine kinase is colocalized with SOX2 positive cells in the dentate gyrus. **a**, DAPI staining (blue) show the lower blade of the dentate gyrus. **b,c**, TK staining (red) and SOX2 positive cells (green). **d**, The merged image of a, b and c. The arrows show TK positive staining colocalized with SOX2 positive cells.
(TIF)

Table S1 Summary of data extracted for the meta-analysis of neurogenesis ablation effects on rodent behavior. The table shows the first author and year of publication for each paper. The study.id field identifies the data set used (since some studies report more than one experiment). This field is used in Figure 7 to identify each study. The table provides data on the variables extracted that were tested to see if they contributed to heterogeneity. These variables are the method of ablation, the amount of irradiation (if used), the rodent species, the test used (EPM: elevated plus maze, MWM: Morris water maze, OF: open field), the number of shocks given, and the time between shock and extinction test (short = less than three days; long = greater than three days).
(XLS)
