# Supplementary figures and images for: Ablating Adult Neurogenesis in the Rat Has No Effect on Spatial Processing: Evidence from a Novel Pharmacogenetic Model
Source: PLoS Genet. 2013 Sep 5;9(9):e1003718. doi: 10.1371/journal.pgen.1003718 (PMC3764151; doi:10.1371/journal.pgen.1003718)

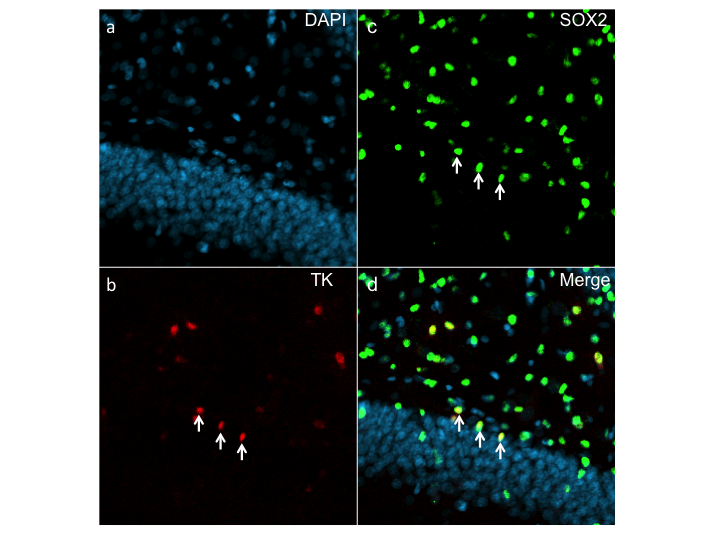

Supplement: Figure S1 — Thymidine kinase is colocalized with SOX2 positive cells in the dentate gyrus. a, DAPI staining (blue) show the lower blade of the dentate gyrus. b,c, TK staining (red) and SOX2 positive cells (green). d, The merged image of a, b and c. The arrows show TK positive staining colocalized with SOX2 positive cells. (TIF) [file pgen.1003718.s001.tif]
